# Workplace Bullying among Managers: A Multifactorial Perspective and Understanding 

**DOI:** 10.3390/ijerph110302657

**Published:** 2014-03-04

**Authors:** J. Antonio Ariza-Montes, Noel M. Muniz R., Antonio L. Leal-Rodríguez, Antonio G. Leal-Millán

**Affiliations:** 1 Universidad Loyola Andalucía, C/Escritor Castilla Aguayo, 4, Córdoba 14004, Spain; E-Mail: noelmunic@yahoo.com; 2 Universidad Loyola Andalucía, C/Energía Solar, 1,Sevilla 41014, Spain; E-Mail: alleal@uloyola.es; 3 Universidad de Sevilla, Av. Ramón y Cajal, 1, Sevilla 41018, Spain; E-Mail: aleal@us.es

**Keywords:** workplace bullying, working conditions, regression model, European Working Conditions Survey-2010, satisfaction, work stress

## Abstract

The aim of this paper is to study certain factors that may be determinant in the emergence of workplace bullying among managers—employees with a recognized and privileged position to exercise power—adopting the individual perspective of the subject, the bullied manager. Individual, organizational, and contextual factors integrate the developed global model, and the methodology utilized to accomplish our research objectives is based on the binary logistic regression model. A sample population of 661 managers was obtained from the micro data file of the 5th European Working Conditions Survey-2010 (European Foundation for the Improvement of Living and Working Conditions) and utilized to conduct the present research. The results indicate that the chance for a manager to refer to him/herself as bullied increases among women that hold managerial positions and live with children under 15 at home, and among subjects that work at night, on a shift system, suffering from work stress, enjoying little satisfaction from their working conditions, and not perceiving opportunities for promotions in their organizations. The present work summarizes an array of outcomes and proposes, within the usual course of events, that workplace bullying could be reduced if job demands were limited and job resources were increased. The implications of these findings could assist directors/general directors in facilitating, to some extent, good social relationships among managers.

## 1. Introduction

### 1.1. Power in the Organization and Bullying

Organizational structure and organizational culture are not neutral. In this sense and in the context of profit maximization and centrality of exploitation during the work process, workplace bullying would be, from a Marxist perspective, normal in the day-to-day management, which forces to pay special attention to sources, meaning, and dynamics originated by power inequalities in the workplace [1].

Although rare, the presence of those critical perspectives in the organizational analysis and, particularly, some labor achievements in developed countries have permitted awareness of certain limits in the organizational demands, acknowledging workplace bullying as a reiterated and irrational behavior that causes damage or has the potential to do it [2,3]. Today, some authors even consider workplace bullying as one of the most devastating problems for employees, to the extent of considering it as probably the severest ways of stress at work [4,5,6]. 

Bullying has a detrimental effect on both individuals and organizations; outlays to the organization are high when lost productivity, turnover, distraction of witnesses, and emotional and physical health costs of targets are measured. This number exponentially increases when a potential lawsuit for unjust dismissal or workers compensation and disability are added. Costs that are harder to calculate but are negatively impacted are reduced work quality, errors, absenteeism, or poor reputation and customer relationships that result from loss of work focus and commitment [7].

In the context of Industrial Psychology, international research has shown a growing interest in workplace bullying [8]; more precisely, the investigation into bullying experienced by managers, although still in its infancy, has become part of a modest but emerging body of literature on this realm [9]. The aim of this paper is to study certain determinant factors for workplace bullying that might affect this group of employees with a recognized and privileged position to exercise power—managers—adopting the individual perspective of the subject—the manager bullied—regardless of the frequency or duration of the action: managers, as common subjects, are in the most personal position to judge whether they are bullied at work or not because if they perceive they are, the adverse effects that arise from the experience would manifest independently of the presence of solid fundamentals in that perception. 

Having said that, we begin our paper conceptualizing and defining the workplace bullying phenomenon, followed by the description of some pertinent research on bullying to managers and a revision of the main empirical findings that will allow us to establish a global model of workplace bullying. Later, the most relevant results of the empirical study obtained through a logistic regression analysis are presented, and finally, the discussion and conclusion sections are elucidated.

### 1.2. Delimitation and Effects of Workplace Bullying

The concept of workplace bullying, that in principle may seem plain, has in practice many nuances that should be analyzed. On the one hand, any study on this phenomenon should start from the basic premise of difficulty involved in providing any digit on the prevalence of this event since the revision done by Zapf *et al.*, which reveals that the range may fluctuates between 5% and 30% [10]. In fact, the prevalence of bullying varies significantly from one country to another and even within the same country. In Europe, prevalence studies have reported workplace bullying rates of approximately 4%–10% [10], although approximations may vary depending on method of measurement and estimation [11,12]. 

On the other hand, different concepts have been explored and related to bullying by researches [13]: Intimidation, harassment, victimization, aggression, emotional abuse, psychological harassment, or mistreatment at the workplace, among others [14]. This definition proliferation has hindered the ability to conceptualize the phenomenon of workplace bullying in a clear and consistent way, and obscured effective collaboration among researchers and practitioners [15,16,17,18].

Based on the prevailing academic paradigms, this concept entails a type of interpersonal aggression at work, characterized by features of intensity, frequency, duration, and power disparity [3,19,20]; according to Lutgen-Sandvik *et al.*, intensity specifies the number of multiple negative acts [21], while a weekly frequency of these acts over a period of six months has been considered as an operational definition for bullying: severe cases of workplace bullying are differentiated from a less intense bullying, as for example some kind of stress at work [3,20,22,23,24,25]. Furthermore, researchers often apply a six-month duration criterion to differentiate bullying from other negative lower intensity acts [4,25,26,27]. Nevertheless, the first bullying act at work implies a breaking point that will affect both bully and victim from then on. It does not need to be reiterated in order to produce its negative effects [28]. In this vein, our approach is consistent with Leymann’s research, which characterizes mobbing as a unique negative act. Finally, power disparity between bully and victim is central for the definition of bullying [3], that is to say, those who are bullied feel unable to protect themselves, as they have little chance of taking revenge on their aggressors [29].

Workplace bullying encapsulates a series of systematically negative acts that derive into social, psychological, and psychosomatic problems for the victim [3]. Therefore, although definitions tend to focus on persistence and duration as key criteria of the phenomenon, the present paper disagrees on this perspective, as workplace bullying has a strong psychological component. In fact, an essential condition for bullying is that the act must be perceived as hostile by the target [30,31]: During these sort of incidents, it is perceived an imbalance and misuse of power between the perpetrator and the target, an inadequate support, and a target’s inability to defend him/herself from such aggression [3,30,31,32,33], as well as a need to cope with negative and constant social interactions [33], physical or verbal badgering, insulting remarks [3], and intense pressure [34]. From this viewpoint, noxious effects of workplace bullying (anxiety, depression, absenteeism, lack of organizational commitment…) would actually spring out the very moment the target perceives the hostile conduct, independently of persistence or duration of the act; from that moment on, worker’s behavior will change substantially. As Einarsen and Raknes point out, victims’ resentment will affect performance at work causing an unpleasant work environment [35]. In this regard, Hoel *et al.* suggest that workplace bullying reduces organizational efficiency, as it decreases employee morale, productivity, and motivation, at the time that absenteeism and employee turnover increase [36].

The harmful effect of workplace bullying have been recently identified in different levels of the organization, demonstrating that bullying influences can be conveyed to various directions; that is, horizontally within the same level (among co-workers) [25,37], upwardly from a lower to a higher level (from subordinates to managers), and downwardly from a higher to a lower level (from managers to subordinates) [38]. Specifically, there is an escalating amount of studies on upwards bullying—as opposed to downwards bullying—that illustrates how subordinates in an organization can bully constantly on a senior person or an authority [37,39]. In a research conducted by Branch [37], utilizing an unstructured interview that involved 16 managers within a public organization, it is revealed that all of the interviewees have either personally experienced or witnessed upwards bullying within that organization. According to Branch *et al.* [39] upward bullying, in general terms, has to be considered a multidimensional concernment that includes a problematic work environment, noted conflicts within the workgroup, inappropriate expressions of emotion, and power imbalances. Beside, while the gender incidence of aggressors and victims of bullying is generally equal, there is some evidence that the targets of upward bullying are more often women [40,41].

Despite these investigations, it has been obtained very limited information about the perpetrators of bullying and their perspective on bullying, as there are both practical and ethical considerations that make it difficult both to approach and study this group [42]. With respect to subjects that hold a managerial position, managers might behave both destructively and constructively, exhibiting dissimilar behaviors and combinations of behaviors in relation to the subordinates, who again may react in a particular way [42]. 

### 1.3. Factors Influencing Workplace Bullying

#### 1.3.1. Towards a Multifactorial Understanding of Workplace Bullying

Due to the severe negative consequences of workplace bullying on mental health and well-being of employees, and, hence, on the performance of the organization, it is vitally important to understand the factors that contribute to the emergence and development of this phenomenon [43]. In this sense, there is a research trend leaded by psychology that focuses on victim and/or bully pathology. From a humanist perspective, this dominant line of thought highlights workplace bullying at the individual level, producing much research linked with psychological effects and therapeutic practice in support of victims. However, the bullying research field has always provided a sufficiently broad approach, with groups of researchers considering the influence of micro-organizational factors—such as role conflict, leadership, political aspects, or organizational culture—as relevant [3].

As a result, research on workplace bullying has evolved towards a multi-causal understanding; in this line, Hoel and Cooper identify five main areas of analysis depending on where the main focus could be: On the individual, on the social interaction between two (or more) people, on group dynamics, on working environment (dealt with in our research), or on a wider context at the level of organization, society, and political scene [44]. Nevertheless, most researchers agree that workplace bullying turns out from the result of the interaction of some factors that manifest in the individual, organizational, and contextual ambits [17,45,46,47].

The following section will first offer a compilation of the main empirical findings on workplace bullying and a synthesis, later, of the hypotheses that have configured the theoretical model for this research, which could be used to derive some specific characteristics for the case of employees with managerial responsibilities.

#### 1.3.2. Workplace Bullying Individual Factors

Some personal characteristics of victims could constitute, in principle, a workplace bullying antecedent. In fact, first studies suggest that those employees who suffer conflict at work used to experiment it in other contexts such as within the couple relation, in the family, and with friends [48]. The perspective of individual antecedents to workplace bullying has been a controversial issue as “blaming the victim” may result [49]. Yet, research on a personality inclined to bullying is far from being conclusive [50]. Most researchers conclude that a personality predisposed to play the role of victim or bully may not exist [20,51].

However, some studies have attempted to identify certain individual factors—gender, age, and seniority—that could increase the risk of becoming victim or bully [47,51]. Presence or absence of these factors affects bullying ratio [52]. This happens as bullies estimate costs and potential profits of bullying in terms of specific features of victims, as certain groups (e.g., women or junior employees) seem to be more vulnerable. 

One of the key factors that could be used to study bullying at the individual level is gender. However, results of empirical studies that have analyzed this aspect do not seem to be very conclusive. Thus, some authors have observed a higher frequency of bullying among women [4,5,45,53,54], while other large-scale studies conclude that, except for sexual harassment, both men and women are equally prone to be bullied at work [9,31,55,56,57,58].

In view of the aim of our paper, it is especially relevant the research conducted by Veale & Gold, which reveals that women in management positions are more vulnerable to bullying [59]. According to the authors, the explanation can be found in still predominant sexist attitudes as well as in structural barriers that inhibit women’s careers as compared with their male colleagues’.

In any case, Einarsen *et al.* suggest that gender differences found by some researchers are, in fact, consequence of the discrimination that both genders may suffer due to their position at work [3]. From this perspective, incidence of bullying would correspond to the fact of being part of a minority at work, independently of the gender of this minority.

Findings related to another personal factor such as victim age do not show a clear relation. Thus, Rayner reports that bullying victims are normally under 25 [56]; later, Hoel and Cooper find that young people are more likely to experiment a greater level of bullying than older employees [55]. However, Einarsen *et al.* [60] just report the opposite situation, and Einarsen and Skogstad [31] come across with a higher incidence of bullying among senior employees. This same conclusion is found in later research [61,62].

#### 1.3.3. Workplace Bullying Organizational Factors

Considering organization as a whole is essential to understand the phenomenon of bullying, as it is quite difficult to imagine the labor context as independent or not influencing, and thus, triggering bullying at work. Therefore, although first studies focus mainly on psychological characteristics of bullies and their victims, since the 90s, researchers have considered with higher emphasis the influence of some labor and structural characteristics of the organization. We will present, hereafter, a brief bibliographical revision of the main studies that have analyzed the relation among some internal dynamics—such as job stability, job design, or human resources practices—and workplace bullying.

##### Job Stability

The level of employee stability at work can influence the degree of vulnerability towards bullying, not only because less stable and eventual employment is common among lower-status professional jobs, but also because insecurity reduces the power of employees (no matter the level held) *vis-à-vis* their superiors. Empirical research carries out among employees of a university centre sustains that flexible working arrangements contribute to the prevalence of bullying [63]. This circumstance is due to the fact that flexibility context implies less job security, fewer opportunities for socialization, and less time for conflict resolution, which could contribute indirectly to aggression and bullying [45]. In fact, one of the reasons given to explain the increase of bullying in 21st century organizations is precisely the organizational restructuring process, which has enlarged the power gap between management and employees [64,65] with a high rate of outsourcing. Workers are more inclined to feel intimidate in those chaotic and unpredictable environments marked by insecurity, role conflict, or tension [66,67].

Against these antecedents, it could be assumed that rates of bullying among employees with temporary contracts would be higher than that among their colleagues with permanent contracts. However, Kivimäki, Elovainio, and Vahtera do not observe any difference between them, neither between full-time and part-time employees [68]. In reference to this aspect, research results are also conflicting. While Baron and Neumann find a positive relation between part-time and bullying [69], Hoel and Cooper observe that same relation among full-time employees [55].

##### Intrinsic Characteristics of the Job

Empirical research on the relation between workplace bullying and intrinsic characteristics of the job position is also extensive. Prior studies have identified and examined many of these elements, such as workload [46,55,60], control [4,57,70,71,72], role ambiguity [60,73], role conflict [60,74], leadership behavior [57,60], social support from co-workers and supervisors [4,75], social climate [4,55,57,60,71,76], and organizational change [55,77,78,79].

A large investigation conducted in United Kingdom among 5,200 people reveals that bullying victims, as compared with non-bullied, suffer from workload, rarefied working environment, greater organizational changes, unsatisfactory relations at work, and greater intention to resign [55]. Likewise, in a study on Norwegian employees, Einarsen *et al.* discover a significant correlation between the variables mentioned above and bullying: workload, control, role ambiguity and role conflict, leadership behavior, social climate, and organizational change [60]. In a similar line, Salin notices that bullying correlates with politized and competitive organizational climate, and more slightly with workload [46]. Vartia’s research identifies, as significant variables of bullying, the precarious social climate, internal communication problems, and the prevalence of a competitive work atmosphere [57]. In the same way, with a sample of 400 employees from five Swedish organizations, Hansen *et al.* find a reverse correlation between bullying and support given to employees by their colleagues and their superiors [75].

Bowling and Beehr’s meta-analysis—carried out over a total of 90 studies on bullying published between 1987 and 2005—contributes with some coherence to the investigation on this phenomenon, compiling and organizing the empirical research existing at that time [50]. Relating to characteristics of job position, these authors inform that bullying tends to disclose in work environments where other stressors, such as role conflict (r = 0.44), role ambiguity (r = 0.30), overload (r = 0.28), and work limitations (r = 0.53) are present. Likewise, they confirm that autonomy at work is negatively associated with bullying (r = −0.25).

Further organizational variables studied for their potential relation with bullying are monotony, complexity, or teamwork. Zapf *et al.*’s research evidences that monotonous and repetitive tasks are more usual among bullying victims [4]. In a later investigation, Zapf does not corroborate any association between bullying and work complexity [18]. Zapf *et al.* state that in those activities where teamwork is present, bullying among equals is more likely to occur [4]. According to these authors, the climate generated by these groups contributes to the search of scapegoats, generally among less powerful members, to whom team aggressiveness is targeted.

Some investigations reveal connections between bullying and other individual perceptions on the organization, such as job satisfaction and commitment. In the first case, job dissatisfaction constitutes another phenomenon related to bullying [71]. Regarding the second case, as bully victims are affected emotionally, this phenomenon is necessarily linked to affective commitment. Several authors point out a negative relation between the two variables [55,80]. Moreover, employees highly committed with their organizations could be more vulnerable to stressors in the work environment, due to precisely the emotional link with the organization [81].

##### Occupations and Bullying

Scientific literature contains a variety of studies on bullying related to subjects that hold specific types of occupations. Some studies suggest that 44.0% of nurses have been bullied during the course of their working activities [82]. Other occupations in which there is evidence of bullying include restaurant employees [83], teachers [84], university professionals [52,85,86], business professionals [23], transportation workers [86], and police officers [87].

There are some studies that have examined as well multiple occupations in the same research: Blue-collar workers, clerks and service workers, associate professionals, managers, and professionals [40,88].

Indeed, the perspective of the current study focuses on a privileged group of employees within the company structure: managers. The incidence of workplace bullying among managers is a topic scarcely touched upon in scientific literature. Using a sample of 512 managers in the UK, Woodman and Cook obtain interesting results [89]; to this respect, 39.0% of the respondents report having been bullied in the past three years. Bullying affects individuals at all management levels. Middle managers appear slightly more likely to be affected, with 49.0% reporting having been bullied in the past three years. This may reflect the phenomenon known as management squeeze: where middle managers can be subjected to particular pressures if they are required to implement unpopular policies as a result of decisions having been taken at more senior levels. Directors are least likely to suffer from bullying, but 29.0% still report having been bullied over the previous three years. Further analyses show that these circumstances reflect significant differences between the average scores for private and public limited companies, and organizations in the public sector where a higher level of bullying is reported.

In a previous study, Ariza *et al.* identify different factors that seem to favor the emergence of bullying among managers [90]; according to this research, the probability for a manager to be bullied increases with job insecurity, and bullying is found to a greater extent among people dissatisfied with their jobs and salaries; among those who work predominantly in the public sector—especially those who are not partnered—and among those whose activity is very emotionally demanding.

##### Human Resources Practices

Through their policies, culture, and practice, organizations can originate a promising breeding ground for the appearance and development of bullying. In this line of thought, Bowling and Beehr indicate that personal traits of victims could be found in the origins of bullying, but these victims can consider organizational climate and practices of Human Resources partially responsible of it (recruitment, formation, and remuneration schemes) due to the effects on their jobs (presence of other stressors and presence of bullies) [50]. Roscigno’s research examines thoroughly the incidence of remuneration schemes, pointing out that workers who receive low payment are likely to be exposed to bullying, while well-paid employees are usually better protected as their professional situations put them closer to their superiors [91].

Güneri goes further when he indicates that the most important reason for bullying lies on organizational factors, such as compensation schemes and labor agreements, job position design, culture and organizational climate, leadership and organizational changes, or sector dynamism. We will deal with this last aspect in the following section [92].

#### 1.3.4. Contextual Factors in Workplace Bullying

In addition to factors related to internal dynamics of organizations, bullying rates can also be affected by the context in which the organization operates; a context that could be characterized by the sector of activity, nature, or size of the organization.

Research on this subject reveals that bullying is more frequent in the service sector, especially in health, public service, education, and financial services [54,70]. Leymann argues as well that the most common bullying occurs in health, especially among nurses, due to work overload and to the double supervision they receive (by doctors and chief nurses), breaking the Unity of Command Principle [58]. Supporting this argument, Yildirim and Yildirim evidence that 87% of nurses in Turkey are submitted to some form of bullying, especially those in the public sector [93].

High bureaucracy, existence of very strict norms, and high level of job security generate a pertinent environment for the development of bullying, as this environment makes the bully invisible and the victim less likely to resign [23]. In this sense, Zapf *et al.* offer a summary of European studies concluding that in the public sector—public service, health, education, and public assistance—the prevalence of bullying is higher than in the private sector [10]. A similar conclusion is obtained by Hoel and Cooper in the United Kingdom [55], who illustrates a higher incidence of bullying in public services such as education or correctional assistance, and a lower prevalence in the retail market and in the industrial sector. Likewise, Soares’ research shows that 4.4% of the employees in Public Health and Education Services are bullied by their patients or students [94].

LaVan, Katz and Jedel consider that public sector employees are dissimilar in their employment relationships, which may lead to a distinct form of workplace bullying [14]. Although there are some studies that suggest that bullying may be greater in public sector [10,23,55,95], LaVan, Katz and Jedel think that this actually happens because many public sector jobs have a great deal of emotional labor rather than instrumental roles [10,14,96]. Public sector employees are likely to have a special type of employment status in that they are typically protected by civil service rules and regulations, may well have unions representing them and internal grievance procedures, and/or are likely to be covered by statutes that provide for protection against retaliation for whistle blowing.

## 2. Methods

### 2.1. Hypothesis for Modeling Workplace Bullying among Managers

As indicated in the introduction, the object of the present investigation is to examine certain determinant factors that seem to be related to the appearance of workplace bullying among managers. In this sense, the prevalence of this deleterious experience is expected to be higher among lower-status professional employees, as there is a reverse relation between the possibility of being a victim and the position in the organization [66]. Therefore, managers should have a perception of bullying lower than those employees with no managerial responsibilities. In this regard, Salin’s research, with a sample of professional employees, reveals that only 2.0% of managers have experimented bullying, while 17.5% of employees have suffered it in the last 12 months [23]. However, these results are not decisive, as other empirical studies point out that the ratio of bullying victims happens to be similar among employees, middle, and senior managers [26,35].

As studies on bullying among managers are scarce and the most relevant findings focus on those investigations that discuss this problem in general terms, for the present investigation our assumptions will be based on a broad perspective, assuming initially a model similar to that observed in regular employees, independently of the responsibility held in the organization.

Under this premise, the hypotheses to be contrasted in our empirical study are the following: 

(1) At the individual level, one personal factor is considered determinant for workplace bullying, therefore: 

*Hypothesis 1*: Women in management positions are more vulnerable to bullying.

(2) At the organizational level, we set up the following hypotheses: 

*Hypothesis 2*: The more senior, the less likely to be bullied.

*Hypothesis 3*: The greater the job insecurity, the more likely to be bullied.

*Hypothesis 4*: The probability of being a bullied manager increases when there are poor working conditions (night work, shift work, or job stress).

*Hypothesis 5*: Unsatisfied managers are more likely to be bullied.

(3) Finally, at the contextual level, we formulate the following hypothesis:

*Hypothesis 6*: Managers in the service sector and in public institutions are more likely to be bullied.

Insofar as the model confirms these hypotheses, we could confirm the existence of a specific profile of bullying to an employee with managerial responsibilities, or, on the contrary, the prevalence of the same pattern described by the general models on bullying in the scientific literature. 

### 2.2. Empirical Study

#### 2.2.1. Methodological Design and Data

As we pointed out in the previous section, workplace bullying is a phenomenon whose causes have to be found in different personal, organizational, and social factors. This is the approach that leads the empirical research dealt with in this section, in which the methodological explanation is addressed in accordance with its multidimensional character, the source of used data, the nature of variables, and the obtained results.

The methodology utilizes for the fulfillment of our objectives in this paper is based on the binary logistic regression model, a specific type of dichotomous response regression model. This statistical technique determines the probability of the occurrence of an event—to feel bullied in this case—compared to the probability of the occurrence of the opposite event.

Data used in this research have been obtained from the 5th European Working Conditions Survey, carried out in 2010 by the European Foundation for the Improvement of Living and Working Conditions. The survey provides insight into the working environment and employment situation throughout the 27 EU Member States as well as in Turkey, Croatia, Norway, Macedonia, Montenegro, Albania, and Kosovo. The target populations under study are those aged 15 years and over (16 and over in Spain, the UK, and Norway) who are employed and reside in the country being surveyed. The sample is a multi-stage, stratified, random sample. The total number of interviews in 2010 is 43.816. 

In light of the objectives that sustain this investigation, we obtain a sub-sample of 661 managers and middle managers, of which 49.5% report feeling bullied at work, while 50.5% admit not feeling like being bullied. All the subjects of the sample are senior or middle managers, from the public sector (23.0%) and private sector (77.0%). 64.1% are men and 35.9% women. The average age of the surveyed is 43.14 years (43.62 in men and 42.51 in women). Finally, 5.8% declare not having formal qualifications or having just completed primary education; 58.7% state having finished secondary education while 35.5% completed studies at university.

#### 2.2.2. Used Variables

##### Dependent Variable

Two different approaches have been mainly used in the bullying research when questionnaires have been used: The self-labeling approach and the operational approach. The limitations and benefits of these methods are discussed in Nielsen *et al.* [97] and amply argue in the discussion section of this paper. The dependent variable of this study is bullying at work. Respondents are asked only one question on their individual perception regarding this topic: *Over the past 12 months, during the course of your work have you been subjected to bullying/harassment?* Bullied senior and middle managers are codified as 1, while those who do not feel bullied are codified as 0. 

##### Independent Variables

We consider workplace bullying as a complex phenomenon that results from the interaction of work environment variables and individual factors. Having into account prior studies on workplace bullying, we employ three sets of independent variables grouped into three categories: factors at the personal and familiar level, working conditions, and finally organizational contextual factors. The codification of variables to be considered is presented in Table 1.

**Table 1:** Explanatory variables: coding and frequency

Variable and Coding	Frequency			
	Value 0	Value 1	Value 2	Value 3
**1. Personal and familiar level**				
Gender (0: male; 1: female)	424	237		
Age (0: 15–24; 1: 25–39; 2: 40–54; 3: 55 or over)	21	231	302	98
Education (0: higher education; 1: secondary education or lower	318	340		
Status (0: partnered; 1: single)	452	206		
Children under 15 at home (0: Yes; 1: No)	238	423		
Children of 15 or older at home (0: Yes; 1: No)	181	480		
**2. Working conditions**				
Seniority (0: Up to one year; 1: more than 1 up to 5; 2: more than 5 up to 10; 3: more than 10 years)	29	196	137	286
Type of contract (0: An indefinite contract; 1: A temporary contract)	430	47		
Working hours (0: Less than 20 hours; 1: 20 to 40 hours; 2: More than 40 hours)	11	382	242	
Work at night (0: No; 1: Yes)	474	173		
Work on Sundays (0: No; 1: Yes)	412	238		
More than 10 working hours a day (0: No; 1: Yes)	313	310		
Working day (0: Full time; 1: Part time)	582	53		
Shift work (0: No; 1: Yes)	546	110		
Capacity to decide timetable (0: Flexibility; 1: No flexibility)	374	279		
Harmony between working hours and personal matters (0: Yes; 1: No)	479	178		
Monotonous tasks (0: No; 1: Yes)	367	290		
Complex tasks (0: Yes; 1: No)	473	184		
Rotating tasks (0: Yes; 1: No)	330	325		
Team work (0: Yes; 1: No)	446	209		
Autonomy on the content (0: Yes; 1: No)	588	68		
Autonomy on the pace of work (depending on people) (0: No; 1: Yes)	362	240		
Autonomy on the pace of work (depending on automated systems) (0: No; 1: Yes)	515	143		
Emotional involvement (0: Yes; 1:No)	442	210		
Work stress (0: No; 1: Yes)	130	524		
Working condition satisfaction (0: Yes; 1: No)	519	139		
Payment satisfaction (0: Yes; 1: No)	333	319		
Likely to be dismissed (0: No; 1: Yes)	492	125		
Promotion opportunities (0: Yes; 1: No)	304	322		
Motivation (0: Yes; 1: No)	412	212		
**3. Organizational context **				
Sector/Industry (0: agriculture; 1: industry; 2: construction; 3: services)	9	84	45	510
Type of sector (0: Private; 1: Public)	470	140		
Size (0: Micro enterprise (1-9 employees); 1: Small enterprise (10-49 employees); 2: Medium-large enterprise (50+ employees)	257	176	215	

## 3. Results

### 3.1. Bivariate Analysis

The main objective of this research is to examine certain potential determinants that might explain the emergence of workplace bullying amongst senior and middle managers. To this end, we use an analysis of contingency table and a Pearson’s chi-square test in order to examine the bivariate relationship between the dependent variable—to feel bullied or not—and a set of independent variables grouped into the three categories mentioned above. This estimate assumes a preparation for subsequent multivariate analysis, as the logistic regression model should only include those independent variables with a statistically significant predictability.

The application of Pearson’s contrast at a 0.05 level of significance leads us to exclude from the analysis some variables initially under consideration; first, at the individual level, those variables related to the age of respondents (Sig. 0.658) and to the presence of children above 15 at home (Sig. 0.361) are discarded. Second, at the organizational level, working conditions related to seniority (Sig. 0.163), type of contract (Sig. 0.283), working hours a week (Sig. 0.261), working day—full-time or part-time—(Sig. 0.383), task monotony (Sig. 0.440), team work (Sig. 0.275), autonomy on the pace of work depending on people (Sig. 0.507), and autonomy on the pace of work depending on automated systems (Sig. 0.069) are also excluded. Finally, at a contextual level, we discard the variables related to sector or industry (Sig. 0.632) and to size (Sig. 0.137).

### 3.2. Multivariate Analysis

Following the initial analysis, we present a logistic regression model to determine to what extent the different categories of variables used in this investigation can explain bullying. To prove the effect of every group of variables, we reproduce up to three different models where the addition of each block is treated as a new separate model. To estimate the model, we opt for a step forward method using all the predicting variables of each model to assess the most efficient variable combination in the explanation of workplace bullying to senior and middle managers.

#### 3.2.1. Incidence of Personal and Familiar Factors (Model 1)

As stated above in the literature review, during the last few decades several investigations have pointed out that possible antecedents of bullying might range from organizational factors to personality features [18,47]. In this sense, we can see in Model 1 of Table 2 that female managers, with lower education, who are not living with children under 15 at home perceive bullying to a greater degree. All the findings presented are significant at 1% level. Therefore, the probability of being bullied at work decreases among male managers with higher education who are living with children under 15 at home. For this level of significance, the logistic regression model indicates that living as a couple it is not related—neither positive, nor negatively—to bullying perception.

**Table 2:** Logistic regression: factors that determine workplace bullying.

Variables	Odds Ratios		
	Model 1	Model 2	Model 3
**1. Factors at a personal and familiar level**			
Gender (0: male; 1: female)	0.600	0.471	0.545
Education (0: higher education; 1: secondary or lower	0.331	n.s.	n.s.
Status (0: partnered; 1: single)	n.s.	n.s.	n.s.
Children under 15 at home (0: Yes; 1: No)	0.524	0.554	0.653
**2. Working conditions**			
Work at night (0: No; 1: Yes)		0.701	0.706
Work on Sundays (0: No; 1: Yes)		n.s.	n.s.
More than 10 working hours a day (0: No; 1: Yes)		n.s.	n.s.
Shift work (0: No; 1: Yes)		n.s.	0.669
Capacity to decide timetable (0: Flexibility; 1: No flexibility)		0.499	n.s.
Harmony between working hours and personal matters (0: Yes; 1: No)		n.s.	n.s.
Complex tasks (0: Yes; 1: No)		n.s.	n.s.
Rotating tasks (0: Yes; 1: No)		n.s.	n.s.
Autonomy on the content (0: Yes; 1: No)		n.s.	n.s.
Emotional involvement (0: Yes; 1:No)		n.s.	n.s.
Work stress (0: No; 1: Yes)		1.295	1.537
Working condition satisfaction (0: Yes; 1: No)		1.361	1.265
Payment satisfaction (0: Yes; 1: No)		1.247	1.068
Likely to be dismissed (0: No; 1: Yes)		n.s.	n.s.
Promotion opportunities (0: Yes; 1: No)		n.s.	0.545
Motivation (0: Yes; 1: No)		n.s.	n.s.
**3. Organizational context**			
Type of sector (0: Private; 1: Public)			n.s.
Constant	−0.613	−2.835	−3.106
χ^2^ Efficiency test—Added category	25.435	133.84	1.864
Degrees of freedom	3	4	1
Level of significance	0.000	0.000	0.000
χ^2^ Efficiency test—Global Model	25.435	159.275	161.139
Degrees of freedom	3	7	8
Level of significance	0.000	0.000	0.000
% Correct prediction			
Global	58.6	72.5	74.8
Bullied	59.8	74.0	78.4
Non-bullied	57.5	70.4	70.9

Despite this, the impact of each significant variable on the probability of feeling bullied at work is not the same in all the cases, as it is stated by the analysis of confidence intervals obtained for the corresponding odds ratios (see Table 3). In this sense, there is a slightly higher effect on the variable gender, as the probability of feeling bullied among women practically doubles that of men (OR: 1.822), with a confidence interval that varies from 1.312 to 2.530. Meanwhile, the presence of small children at home increases the probability of bullying by 1.689 times (1.393 in managers with lower education).

**Table 3:** Logistic regression: factors that determine workplace bullying (Confidence intervals for the odds ratio of Model 1)

Variables in the Model					Odds Ratios 95% C.I. for OR		
	B	S.D.	Wald	P	OR	Lower	Upper
Gender	0.600	0.168	12.809	0.000	1.822	1.312	2.530
Education	0.331	0.161	4.253	0.039	1.393	1.017	1.908
Children under 15 at home	0.524	0.167	9.900	0.002	1.689	1.219	2.342
Constant	−0.749	0.175	18.401	0.000	0.473		

The contrast statistic applied to assess the validity of the model in general points out that there are enough reasons to accept its validity, that is to say, to affirm that a set of variables—personal and familiar—taken into account in the first model of our research, can satisfactorily explain if a manager is exposed to bullying at work and to what degree. The omnibus test of the model, used for this purpose, presents the following results: Chi Square: 25.435; Sig. 0.0000. However, the suitability of the model to be widely available—considering only personal and familiar variables—is limited, as 58.6% of the considered individuals are classified correctly knowing their real situation (see Table 2) in advance. Moreover, there exists disparity between the percentages related to bullied (59.8%) and non-bullied (57.5%) managers, what suggests that the former are easier to be identified. These results indicate that there are further factors, apart from those presented in this first model, that contribute to explain the perception of bullying at work

#### 3.2.2. Joint Impact of Factors at the Personal-familiar Level and Working Conditions (Model 2).

The second model incorporates personal and familiar variables as well as those related to working conditions—seniority, autonomy, contract type, timetable, *etc.*—enjoyed by the senior and middle managers of our sample. As can be seen in the last rows of Table 2, when these variables are included, the percentage of bullying prediction increases by 13.9 percentage points, from 58.6% to 72.5%. The validity of the global model improves when the group of variables related to working conditions is added, increasing **χ^2^** up to value 159.175 (Sig. 0.0000). The improvement in the general model comes with a higher balance in the predictability between both groups. Therefore, the capacity of generalization for the group of bullied managers rises at 74.0%, while among non-bullied managers reaches 70.4%.

In Table 4 we can see that, when introducing working conditions in a new combined model, the only personal variables that explain workplace bullying are: being woman (OR: 1.602) and having children under 15 and under their care (OR: 1.740). In this sense, senior and middle managers who work at night (OR: 2.016), without the possibility to decide timetables (OR: 1.647), who work in a stressful job (OR: 3.649), who aren’t satisfaction with their working conditions (OR: 3.900), and who are little satisfied with their payment (OR: 3.479) are more likely to feel bullied. The remaining variables—status, shift work, working on Sundays, promotion opportunities, *etc.*—acting together, do not explain the perception of workplace bullying among managers.

**Table 4:** Logistic regression: factors that determine workplace bullying (Confidence intervals for the odds ratio of Model 2).

Variables in the model					Odds ratios 95% C.I. for OR		
	B	S.D.	Wald	P	OR	Lower	Upper
Gender	0.471	0.218	4.675	0.031	1.602	1.045	2.455
Children under 15 at home	0.554	0.218	6.434	0.011	1.740	1.134	2.670
Work at night	0.701	0.241	8.456	0.004	2.016	1.257	3.234
Capacity to decide timetable	0.499	0.211	5.574	0.018	1.647	1.088	2.493
Work stress	1.295	0.301	18.527	0.000	3.649	2.024	6.580
Working condition satisfaction	1.361	0.293	21.631	0.000	3.900	2.198	6.922
Payment satisfaction	1.247	0.211	34.914	0.000	3.479	2.301	5.262
Constant	−2.835	0.357	63.083s	0.000	0.059		

#### 3.2.3. Joint Impact of Factors at the Personal-familiar Level, Working Conditions, and Organizational Context (Model 3)

In the third model, only a variable of the organizational context has been added: the public or private nature of the organization where the manager works. As recommended by the bivariate analysis, two variables of this category have been excluded: size and activity sector.

Table 2 confirms that the effect of these variables on the capacity of generalization of the model increases the percentage of global prediction slightly (from 72.5% to 74.8%): 78.4% in bullied managers and 70.9% in non-bullied managers. Therefore, the inclusion of organizational variables improves the capacity of prediction of the model, as with the validity of the model (Chi-square: 161.139; Sig. 0.000).

The influence of those variables regarding organizational context in the model of workplace bullying introduces some alterations that result in the final model presented in Table 5. Thus, the probability for a manager to feel bullied increases among women (OR: 1.725; CI: 1.092–2.727) with small children at home (OR: 1.922; CI: 1.223–3.020), who work at night (OR: 2.025; CI: 1.178–3.483), on a shift system (OR: 1.951; CI: 1.036–3.676), who suffer from work stress (OR: 4.65; CI: 2.439–8.862), who feel little satisfied with their working conditions (OR: 3.543; CI: 1.911–6.569) and with their payment (OR: 2.908; CI: 1.854–4.562), and who don’t see opportunities for promotion within their organizations (OR: 1.725; CI: 1.087–2.736).

**Table 5:** Logistic regression: factors that determine workplace bullying (Confidence intervals for the odds ratio of Model 3)

Variables in the Model					Odds Ratios 95% C.I. for OR		
	B	S.D.	Wald	P	OR	Lower	Upper
Gender	0.545	0.234	5.451	0.020	1.725	1.092	2.727
Children under 15 at home	0.653	0.231	8.026	0.005	1.922	1.223	3.020
Work at night	0.706	0.277	6.515	0.011	2.025	1.178	3.483
Shift work	0.669	0.323	4.280	0.039	1.951	1.036	3.676
Work stress	1.537	0.329	21.806	0.000	4.650	2.439	8.862
Working condition satisfaction	1.265	0.315	16.119	0.000	3.543	1.911	6.569
Payment satisfaction	1.068	0.230	21.607	0.000	2.908	1.854	4.562
Promotion opportunities	0.545	0.235	5.357	0.021	1.725	1.087	2.736
Constant	−3.106	0.397	61.267	0.000	0.045		

## 4. Discussion

As a result of its negative consequences on mental health and well-being of employees, and hence on the performance of the organizations, the understanding of the factors that favor the emergence and advance of bullying is vital [43]; specially in the development of more effective prevention and intervention tools to remedy this social problem [98,99]. 

In this line, bullying prevalence varies notably from one country to another, even within the same nation; in Europe, for example, even though the inferences may differ depending on the measurement and estimation methods being utilized [11,12], studies of the occurrence of workplace bullying report rates of approximately 4%–10% [10]. 

For the present research, the prevalence rate obtained happens to be significantly higher: 49.5% of senior and middle managers label themselves as bullied in their professions. However, this relation is similar to that described by Woodman and Cook where 49.0% of middle managers report having been bullied in the last three years [89]; comparable but in other professions, Dellasega finds that 44.0% of nurses experience episodes of bullying at some point during their working lives [82]; and more recently Mintz-Binder and Calkins reveal that the 32.8% of program directors affirm having been exposed to bullying—due to the influence of students and faculty—within the last 12 months [9].

These results indicate that the rate of workplace bullying for professionals in managerial positions is larger than the predicted average calculated, with similar parameters, for employees laboring in any other occupational level and sector. Regarding this point, Zapf *et al.* provide an extensive summary of European studies and conclude that the prevalence of bullying results higher in the public sector (e.g., service, health, education, and assistance) than in the private sector [10]. A similar inference is reached by Hoel and Cooper in the United Kingdom, who report a higher incidence of bullying within public services, such as education or correctional assistance, and a lower incidence in the retail and industrial sectors [55]. Similarly, Soares’ research shows that 4.4% of public education and health care employees have been occasionally bullied by their patients or students while completing their daily tasks [94].

A body of literature has emerged describing the possible triggers of workplace bullying within employees and has focused primarily on two areas. The first area pertains to the personal and individual differences among those involved in the bullying incidents, while the second pertains to the characteristics of the surrounding organizational settings in which these circumstances occur. Similar to the present paper, many authors currently embrace the concept that both individual and organizational factors are important to understand bullying behaviors. For instance, researchers have observed that older workers experience significantly less violence than young workers [100,101]. Other characteristics that have been associated with an increased risk of workplace bullying include gender and marital status; a greater percentage of female physicians, for example, fear a potentially violent encounter at work compared to male physicians [102]. Lin and Liu’s study reports that unmarried workers are significantly more likely to experience workplace violence compared to married employees [103]. In the European Union, these results suggest that there are specific sociodemographic features that may influence the phenomenon of workplace bullying. 

Regarding this concern, the current regression analysis outcomes seem to indicate a tendency for female managers (*Hypothesis 1*), who are living with children under 15 at home—at the individual level—to suffer bullying. According to the statistical results, the group described above seems somehow to be in a position of greater likelihood to suffer from bullying in relation to the other identified groups, for example, men with or without children under their care. Additionally, regarding those concrete cases related to female jobholders, one possible reason that might explain the observed statistical predominance of this group could be the presence of certain sexist attitudes in their surroundings, as well as structural barriers that could inhibit women’s careers to a certain extent compared to their male colleagues. If presented, these circumstances could definitely make the subjects of this group particularly more vulnerable and likely to end up as victims of workplace bullying. This could be a promising ground for further research.

Furthermore, some organizational factors are found to increase the odds of workplace bullying against employees. For instance, with respect to working conditions, it has been claimed in recent meta-analyses that there are some specific organizational variables (e.g., workplace bullying antecedents) that are worth noting, such as conflict and role ambiguity [50], work overload, stress, lack of autonomy, and absence of organizational fairness [74]. Zapf *et al.*’s research shows that performing monotonous and repetitive tasks is more common among bullying victims [4]. At the organizational level, this study emphasizes that the propensity for a worker in a managerial position to experience bullying escalates among those who work in poor working conditions, such as working at night and on a shift system, and suffer from job stress (*Hypothesis 4*); they experience a lack of satisfaction due to their working conditions and payment (*Hypothesis 5*), and do not perceive any opportunity for promotion within their organization. 

This unpredictable environment, characterized by insecurity, role conflict, and tension, allows few opportunities for socialization and even less time for conflict resolution; both of these factors may indirectly contribute to the emergence of aggressive behaviors and bullying. Ultimately, a stressful social climate and a precarious work atmosphere create a breeding ground for workplace bullying, as reflected in the results of the present empirical study. 

Finally, seniority (*Hypothesis 2*), job insecurity (*Hypothesis 3*), and type of sector (*Hypothesis 6*) seem to have no effect on workplace bullying among managers. Thus, it could be accurate to say that the contextual variables of an organization do not influence workplace bullying. Given this finding, it is possible to deduce that workplace bullying may be prevalent with the same degree of intensity in both public and private organizations, regardless of their size.

With these outcomes, the present work might contribute, on the one hand, to shed light on certain latent factor that seem to be linked to the appearance of workplace bullying among managers and, on the other hand, to join to the previous literature on this field that has so far validated the prevalence of these variables among bullied subjects and highlighted the extension of this concern to higher levels of organizations. Additionally, we have identified two more factors that have been virtually absent from the bibliographic review; the study has revealed that the probability for a manager to feel bullied increases among those who are living with children under 15 at home -at the individual level—and among those who do not perceive opportunities for promotion within their organizations—at an organizational level.

## 5. Conclusions

Workplace bullying has become a serious and growing problem that affects a significant proportion of professionals. The serious detriments that workplace bullying causes on health, social, and personal stability of employees, and the general performance of organizations have drawn attention to the comprehension of its appearance and progression [43]. In this regard, the present study aims to contribute to the development and implementation of measures to prevent bullying in employees that hold managerial positions within organizations.

The multidimensional model created in the present research is intended to identify senior and middle managers that are prone to being bullied at work as individuals; the study findings have valuable and pertinent implications for institutions, organizations, and corporations that aim to thrive and to enhance organizational performance throughout all the levels. This work provides reasonable evidence that could be of significant benefit in the implementation of human resource policies: Responsible directors/general directors could reduce the organization-wide levels of workplace bullying by adjusting certain working conditions that negatively affect managers who are especially susceptible to being bullied, given their personal characteristics.

This research paper offers an empirical basis for further studies, related to bullying among employees with a recognized and privileged position to exercise power—managers—adopting the individual perspective of the subject—the bullied manager—in Europe. Attracting and retaining the most qualified and experienced professionals has become essential for successful and competitive organizations; corporations are urged to implement strategies oriented toward reducing workplace bullying. Consequently, the labor force has specific traits that should not be ignored.

From a practical standpoint, the present findings could assist directors/general directors in facilitating harmonious social relationships among managers and subordinates. Particularly, the results suggest that limiting job demands and increasing job resources could reduce workplace bullying. Specific attention should be paid to women in managerial positions who feel dissatisfied with their working conditions, as they constitute a group with an increased risk for experiencing bullying.

Despite the significant findings of this study, its intrinsic methodological limitations must be considered. First, the phenomenon of bullying is measured by self-report, which might increase the risk of common method variance, forcing us to assume a corresponding bias in the key variables. Second, by utilizing self-identification without a strict definition, bullying is measured in broad terms, and consequently, there is a risk of overestimating its prevalence, as the respondents could report incidents that would not qualify as bullying according to the researchers’ understanding of the phenomenon [104]. Third, a related methodological problem could be social desirability; previous scholars have analyzed the repercussions of desirability in workplace bullying studies. Given the particular understanding of the phenomenon under investigation, it seems probable that any given prevalence rate would exceed the rates obtained in this type of research, as many of the present victims took a large amount of time to acknowledge and accept that they were subjected to aggression of this nature. This predicament is particularly acute among certain population groups that are considered more vulnerable, such as women, young people, and employees with temporary interrelations. To correct this problem, some authors propose to make use of multi-method data and utilize objective measures that may reinforce workplace-bullying research. Examples of this type of data include managerial reports and scores from third parties (e.g., researchers) [104]. It should be noted, however, that assessing third party scores of workplace bullying without trying to counteract these behaviors raises ethical concerns due to the many negative consequences of workplace bullying for the parties involved, as well as for the work unit and the organization [105]. Fourth, the observed correlations between bullying and the variables analyzed in this study should be pondered cautiously, as the data are cross-sectional and not experimental. Finally, this study represents only a partial perspective of the phenomenon: the point of view of the victim, not of the bully.

Adjusting job demands and improving job resources and conditions may lessen workplace bullying. To this respect, one option could be to orientate general directors about “*internal marketing*” as a way to sell the company culture internally to employees and senior/middle managers and to somehow help prevent these negative organizational experiences [106,107].
